# Association between the rs2910164 Polymorphism in Pre-Mir-146a Sequence and Thyroid Carcinogenesis

**DOI:** 10.1371/journal.pone.0056638

**Published:** 2013-02-22

**Authors:** Wen-Jun Wei, Yu-Long Wang, Duan-Shu Li, Yu Wang, Xiao-Feng Wang, Yong-Xue Zhu, Ya-jun Yang, Zhuo-Ying Wang, Yan-yun Ma, Yi Wu, Li Jin, Qing-Hai Ji, Jiu-Cun Wang

**Affiliations:** 1 Department of Head & Neck Surgery, Cancer Hospital, Fudan University, Shanghai, China; 2 Department of Oncology, Shanghai Medical College, Fudan University, Shanghai, China; 3 Ministry of Education Key Laboratory of Contemporary Anthropology and State Key Laboratory of Genetic Engineering, School of Life Sciences and Institutes of Biomedical Sciences, Fudan University, Shanghai, China; 4 Fudan-Taizhou Institute of Health Sciences, Taizhou, Jiangsu, China; Consiglio Nazionale delle Ricerche (CNR), Italy

## Abstract

**Background:**

Rs2910164, a Single nucleotide polymorphism (SNP) located in the precursor microRNA sequence of miR-146a, is the only MicroRNA sequence SNP studied in papillary thyroid cancer (PTC). Association studies had been performed in US and UK-Northern European populations, but results were inconsistence. This study evaluated the association between rs2910164 and the risk of PTC as well as benign thyroid tumor (BN), and examined the clinicopathological characteristics of PTC and BN for different genotypes.

**Methods:**

This case-control study genotyped rs2910164 in 753 PTCs, 484 BNs and 760 controls in a Chinese Han population. Clinicopathological and genetic data were collected and compared. Multivariate logistic regression was performed to calculate adjusted odds ratios (ORs).

**Results:**

There were no differences in rs2910164 genotype distributions between the three groups. PTC cases with three genotypes (CC, CG, GG) had similar clinicopathological characteristics except the existence of “para-cancer” BN (PTC/BN, P = 0.006). PTC/BN patients were older (P = 0.009), and had smaller cancer lesions (*P*<0.001), lower serum thyrotropin levels (1.82±1.42 *vs*. 2.21±1.74, *P = *0.04), and lower rates of level VI lymph node metastasis (20.8% *vs*. 52.7%, *P*<0.001) and lateral neck lymph node metastasis (11.5% *vs*. 23.0%, *P = *0.011) compared with PTC only. Then we supposed a possible progression from BN to PTC which may involve rs2910164 in and performed a multivariate logistic regression analysis of PTC/BN and BN cases to determine risk factors of this progression. Results showed that the rs2910164 GG homozygote (OR = 2.25, 95% CI 1.22–4.14, P = 0.01) was the only risk factor in this study.

**Conclusion:**

Rs2910164 was not associated with increased risk of PTC and BN in Chinese patients, but may play a latent role in the transformation from BN to PTC.

## Introduction

The incidence of thyroid cancer, the fifth leading cancer in females, is increasing [1]. Papillary thyroid cancer (PTC) is the most common type of thyroid malignancy but its etiology remains largely unknown [2]. The genetic predisposition of PTC has been evaluated in pedigree studies, association studies and genome wide association study [3], [4], [5], [6], [7], [8]. A series of single nucleotide polymorphisms (SNPs) were found to be associated with the risk of PTC in studies with different designs and populations, which indicated that the genetic etiology of PTC was complicated, and the results needed further investigation in independent studies to confirm the associations with PTC risk.

MicroRNAs are small noncoding RNA molecules that function as post-transcriptional suppressors of gene expression, and are involved in almost all cellular processes, such as proliferation, differentiation, apoptosis and metabolism [9]. The role of microRNA in carcinogenesis of many kinds of malignant tumors, including thyroid cancer, has been well established [10], [11], [12]. A SNP located within its mature sequence or within the “seed” region may change its normal functions. The only SNP located in the microRNA sequence studied in PTC is rs2910164 in the precursor microRNA sequence of miR-146a, and an association between rs2910164 and risk of PTC was found in US and European populations [13], [14]. A further functional study of rs2910164 GC heterozygotes showed that GC heterozygotes differed from both GG and CC homozygotes by producing three mature microRNAs that modulated genes mainly involved in the regulation of apoptosis, leading to an exaggerated DNA-damage response in heterozygotes [15]. However, a recent study failed to replicate such a relationship in a white UK population of northern European origin [16]. There were no further studies investigating these conflicting results, especially in populations of non-Caucasian origin.

The genetic predisposition of a benign thyroid tumor (BN) has been little studied, although more than 70% of females develop a BN in their lifetime. The aim of this study was to evaluate the association between the rs2910164 SNP and the risk of PTC and BN, and to determine the clinicopathological characteristics of PTC and BN in patients with different genotypes.

## Materials and Methods

### Study Population

In this case-control study, the case population consisted of 760 PTC cases and 485 BN patients treated at the Department of Head and Neck Surgery, Cancer Hospital, Fudan University, Shanghai, China, from January 2010 to December 2010. All subjects were ethnic Chinese Han and came from Eastern China, including Shanghai, Jiangsu and the surrounding regions. Enrollment criteria including histologically identified diagnosis, no previous surgical or medical treatment of thyroid disease, no history of familial thyroid cancer, and no radiation exposure. The control population consisted of 760 cancer-free healthy subjects recruited from the Taizhou Longitudinal Study in the same period, with the selection criteria including no individual history of cancer or thyroid disease [17]. Each eligible subject was personally interviewed to gather demographic data (such as age, sex and ethnicity) and environmental exposure history, including radiation, smoking, and alcohol consumption. All the control subjects were frequency matched to PTC cases on age (±5 years) and gender. This study was approved by the Ethical Committee of Cancer Hospital of Fudan University and all patients provided written informed consent.

The management of thyroid tumor in the Cancer Hospital, Fudan University was described previously [18], [19]. Briefly, in our hospital, all patients received an ultrasound examination before surgery. Fine needle aspiration and Computed Tomography were not performed routinely. Lobectomy with pathological frozen section examination was routinely performed during the operation. When a malignant diagnosis was reported intraoperatively by frozen section, level VI lymph node dissection was performed. When a benign or undetermined nodule was detected in the contralateral lobe by US, a subtotal lobectomy with frozen section was also initially performed. If malignant lesions were found in both lobes of the thyroid by frozen section, a total thyroidectomy plus a bilateral level VI lymph node dissection was performed.

### DNA Extraction and Genotyping

About 3–5 ml venous blood was collected from each subject. The Qiagen Blood Kit (Qiagen, Chatsworth, CA, USA) was used to extract genomic DNA. Genotyping was performed with the MassARRAY iPLEX platform (Sequenom Inc., San Diego, CA, USA) using an allele-specific matrix-assisted laser desorption/ionization time of flight mass spectrometry assay (MALDI-TOF) [20]. Reagents for genotyping were acquired from iPLEX® Gold Reagent Kit (Sequenom Inc.). Primers for amplification and extension reactions were designed using MassARRAY Assay Design software. Results of the genotyping were read and output by TYPER 4.0 software (Sequenom Inc.). To examine the quality of the results, operators who performed the genotyping assays were unaware of the study group of each sample. Each plate of samples contained at least four internal positive controls of DNA samples randomly selected in the same plate and two negative controls of pure water. No inconsistent or abnormal result was found in positive and negative controls, suggesting a good concordance. **Seven PTC** cases and **one BN** case were excluded from subsequent study because of low quality DNA samples for genotyping.

### Statistical Analysis

Differences in selected variables and Hardy-Weinberg equilibrium were evaluated using the Chi-square test and Student *t* test as appropriate. The odds ratios (ORs) and 95% confidence intervals (CIs) were calculated by univariate and multivariate logistic regression analyses to determine associations between rs2910164 genotypes and alleles and the risk of PTC and BN, and the clinicopathologic characteristics of different population groups defined by histological diagnosis and genotype. In logistic regression analysis, SNP genotypes were categorized by defining dummy variables. A P-value <0.05 was considered statistically significant. All the statistical analyses were performed with the SPSS Software version 12.0 (SPSS, Chicago, IL, USA).

## Results

### Characteristics of the Study Population

As shown in **Table 1**, the final analysis included 753 PTC patients, 484 BN patients and 760 healthy controls. Among 484 BN cases, there were 292 nodular goiter (60.3%), 100 follicular adenoma (20.7%) and 92 cases with both (19%). Distributions of gender were similar in the three groups. No significant differences in age (mean ± standard deviation (SD)) were found between PTC and control groups, while cases with BN were older than those with PTC (48.48±12.19 vs. 46.36±9.24, P = 0.001).

**Table 1:** The age and gender distribution of papillary thyroid cancer (PTC), benign thyroid tumor (BN) and control study population.

Characteritics	PTC(n = 753)	BN(n = 484)	Control(n = 760)	P value
**Age**				
<45	316(42.0%)	177(36.6%)	308(40.5%)	0.161
≥45	437(58.0%)	307(63.4%)	452(59.5%)	0.361
Age (mean ± SD)	46.36±9.24	48.48±12.19	47.32±11.10	0.001^a^
**Gender**				
Male	211(28.9%)	126(26.0%)	220(28.0%)	0.533
Female	542(71.1%)	358(74.0%)	540(72.0%)	

a The similar age distribution were found between PTC and control (*P* = 0.07), while BN cases were older than PTC cases(*P* = 0.001).

### Association between rs2910164 Polymorphism and Risk of Thyroid Tumor

The genotypic and allelic distributions of rs2910164 SNP in PTC, BN and controls are summarized in **Table 2**. The observed genotype frequencies for the SNP agreed with those expected from the Hardy-Weinberg equilibrium in PTC (P = 0.15), BN (P = 0.90) and controls (P = 0.51). No distribution differences of genotype and allele were found between PTC, BN and controls. The allele frequency was similar with that reported by HapMap in a Beijing Chinese Han population (C allele = 0.554, G allele = 0.446).

**Table 2:** Distribution of rs2910164 genotypes and alleles in cases and controls, and their associations with risk of thyroid tumor.

rs2910164	PTC (n = 753)	BN (n = 484)	Control(n = 760)	*P* value a	OR(95% CIs) b PTC v.s. Control	OR(95% CIs) b BNv.s. Control	OR(95% CIs) b PTC v.s. BN
**Genotype**							
CC	294(39.0%)	163(33.7%)	277(36.4%)	0.203	reference	reference	reference
CG	323(42.9%)	241(49.8%)	345(45.4%)		0.88(0.71–1.10)	1.21(0.94–1.56)	0.74(0.58–0.96)
GG	136(18.1%)	80(16.5%)	138(18.2%)		0.93(0.70–1.24)	0.99(0.71–1.39)	0.95(0.68–1.33)
GG+CG	459(61.0%)	321(66.3%)	483(63.6%)		0.90(0.73–1.10)	1.14(0.90–1.45)	0.80(0.63–1.01)
**Allele**							
C	0.60	0.59	0.59	0.595	reference	reference	reference
G	0.40	0.41	0.41		0.97(0.91–1.05)	1.01(0.93–1.10)	0.97(0.89–1.05)

a P value were calculated by Chi square test.

b Adjusted for age and gender by a logistic regression model.

### Characteristics of PTC Patients with Different miR-146a Genotypes

The PTC patients with different rs2910164 genotypes were compared to determine the clinical characteristics of individual genotype carriers. As shown in **Table 3**, patients with the three kinds of genotypes had similar gender, age, tumor size, multifocal tumors, bilateral cancer site, level VI lymph node metastasis and lateral neck lymph node metastasis.

**Table 3:** The clinical characteristics of PTC with different rs2910164 genotypes.

Characteristics	CC Genotype (n = 294)	CG Genotype (n = 323)	GG Genotype (n = 136)	*P* value^a^
**Gender**				
Male	82(27.9)	99(30.6)	30(22.1)	0.173
Female	212(72.1)	224(69.4)	106(77.9)	
**Age**				
<45	129(43.9)	129(39.9)	58(42.6)	0.603
≥45	165(56.1)	194(60.1)	78(57.4)	
**Size**				
≤1 cm	163(55.4)	175(54.2)	83(61.0)	0.394
>1 cm	131(44.6)	148(45.8)	53(39.0)	
**Multifocal**				
Yes	88(29.9)	86(26.6)	35(25.7)	0.555
No	206(70.1)	237(73.4)	101(74.3)	
**Bilateral**				
Yes	53(18.0)	58(18.0)	23(16.9)	0.956
No	241(82.0)	265(82.0)	113(83.1)	
**With (PTC/BN)**				
Yes	26(8.8)	43(13.3)	27(19.9)	0.006
No	268(91.2)	280(86.7)	109(80.1)	
**pN+ (Level VI)**	49.0%	51.1%	41.9%	0.197
**pN+ (lateral neck)**	23.5%	21.7%	16.9	0.305

a P value were calculated by Chi square test.

Among 753 PTC, 96 cases were reported to have concurrent para-cancer BN (PTC/BN, which was defined that PTC lesion and BN were coexisting within at least one lobe of thyroid gland and BN located nearly by or surrounded the cancer site) by pathologists, including 77 nodular goiters, and 19 adenomas. Among the 96 PTC/BN cases, 40 cases was incidental PTC, which were diagnosed as BN by US preoperatively. It is interesting that the frequency of finding para-cancer BN in PTC with CC, CG and GG genotypes were statistically different. (8.8% v.s 13.3% v.s 19.9%, respectively, P = 0.006). To evaluate the differences between any two of these three groups, separate Chi-square tests were performed (P_CC v.s CG_ = 0.079; P_CC v.s GG_ = 0.001; P_CG v.s GG_ = 0.075). The correctedαvalue (the probability of making Type I Error) was 0.0125. The P_CC v.s GG_ = 0.001<0.0125 suggested a significant difference of PTC/BN incidence between CC and GG homozygote carriers.

### Clinical Characteristics of PTC/BN

To further investigate the potential reason for the higher frequency of PTC/BN in GG genotype carrier, the clinical characteristics were compared between PTC alone and PTC/BN. As shown in **Table 4**, patients with PTC/BN were older (P = 0.009), and had smaller cancer lesions (P<0.001), lower serum thyrotropin (TSH) levels (1.82±1.42 vs. 2.21±1.74, P = 0.04) and lower rates of level VI lymph node metastasis (20.8% vs. 52.7%, P<0.001) and lateral neck lymph node metastasis (11.5% vs. 23.0%, P = 0.011). Frequencies of the GG homozygote genotype and G allele were higher in PTC/BN than PTC alone (28.1% vs. 16.6%, P = 0.006 for genotypes and 50.5% vs. 37.9%, P = 0.001, for alleles).

**Table 4:** The clinical and genetic characteristics of PTC with and without para-cancer benign tumor.

Characteristics	No para-cancer BN (n = 657)	Para-cancer BN (n = 96)	*P* value
**Gender**			
Male	188(28.6)	23(24.0)	0.395
Female	469(71.4)	73(76.0)	
**Age**			
<45	288(43.8)	28(29.2)	0.009
≥45	369(56.2)	68(70.8)	
**Size**			
≤1 cm	349(53.1)	72(75.0)	<0.001
>1 cm	308(46.9)	24(25.0)	
**Multifocal**			
Yes	177(26.9)	32(33.3)	0.222
No	480(73.1)	64(66.7)	
**Bilateral**			
Yes	110(16.7)	24(25.0)	0.062
No	547(83.3)	72(75.0)	
**Hashimoto**			
Yes	62(11.0)	8(8.3)	0.594
No	595(89.0)	88(91.7)	
**TSH(mean±SD)**	2.21±1.74	1.82±1.42	0.04
**pN+ (Level VI)**	52.7%	20.8%	<0.001
**pN+ (lateral neck)**	23.0%	11.5%	0.011
**Rs2910164**			
CC	268(40.8)	26(27.1)	0.006
CG	280(42.6)	43(44.8)	
GG	109(16.6)	27(28.1)	
G allele frequency	37.9%	50.5%	0.001

The results presented above suggested that PTC/BN was a special subgroup in which the cancer was relatively indolent (smaller tumor size, less lymph node metastasis). A possible explanation was that the malignant lesions might have originated from a susceptible background of long-lasting benign tumors and remained at an early stage of carcinogenesis. The high frequency of rs2910164 GG genotypes in PTC/BN suggested that the Mir-146a SNP rs2910164 G allele may play a potential important role in progression to PTC.

### Risk Factors for Malignant Transformation in Patients with BN

The mechanism of the transformation from BN to PTC has not been extensively studied, but a high TSH level and Hashimoto’s thyroiditis were regarded as risk factors for PTC as demonstrated in a series of reports [21], [22], [23], [24], [25], [26]. In the current study, Hashimoto’s thyroiditis was found in 5.8% (25/484) of BN and 10.6% (80/753) of PTC (P = 0.004), and the TSH level (mean ± SD) was higher in PTC than in BN (2.11±1.67 vs. 1.71±1.27, P = 0.001).

To further evaluate the potential risk factors associated with this possible progression from BN to PTC, the clinicopathologic and genetic features of BN cases and 96 PTC/BN cases were compared. Univariate analysis showed that the GG genotype (P = 0.026) and G allele (P = 0.020) distribution of rs2910164 were associated with carcinogenesis on a background of BN, whereas gender, age, presence of Hashimoto’s thyroiditis, and serum TSH level had no significant effects (**Table 5**). A multivariate logistic regression analysis was employed to adjust for confounding factors and confirmed that the GG homozygote (OR = 2.25, 95%CI: 1.22–4.14) was the only independent risk factor associated with the hypothetical malignant transformation in patients with BN.

**Table 5:** The clinical and genectic characteristics of benign tumor (BN) and PTC with para-cancer benign tumor (PTC/BN).

Characteristics	BN (n = 484)	PTC/BN (n = 96)	*P* value^a^	Multivariate regression	
				Adjusted OR	P^b^ value
**Genotype**			0.026		
CC	163(33.7)	26(27.1)		reference	
CG	241(49.8)	43(44.8)		1.18(0.69–2.01)	0.542
GG	80(16.5)	27(28.1)		2.25(1.22–4.14)	0.010
G allele	41.4	50.5	0.020	–	
**Gender**			0.679		
Male	126 (26.0)	23(24.0)		reference	
Female	358(74.0)	73(76.0)		1.05(0.63–1.77)	0.842
**Age**			0.199		
<45	177(36.6)	28(29.2)		reference	
≥45	307(63.4)	68(70.8)		1.42(0.87–2.30)	0.157
**Hashimoto**			0.345		
Yes	28(5.8)	8(8.3)		reference	
No	456(94.2)	88(91.7)		1.23(0.52–2.91)	0.63
**TSH(mean±SD)**	1.71±1.27	1.99±1.99	0.074	1.14(0.98–1.31)	0.073

a P value were calculated by Chi square test.

b Adjusted for age and gender by a logistic regression model.

## Discussion

The SNP rs2910164 is located in the stem structure of human pre-mir-146a and causes a C:U mismatch from a G:U pair between the sequence of mature mir-146a and its passenger strand [13]. Inconsistent results were reported for an association between rs2910164 and PTC risk in two independent studies [13], [16]. In the current study, there were no significant differences in distribution of genotypes and alleles of rs2910164 observed in 753 PTC cases, 484 BNs and 760 controls (**Table 2**). Distinct rs2910164 genotype distribution frequencies were found between the current results and those reported by Jazdzewski et al (for controls, GG: 18.2% vs. 58.4%; GC: 45.4% vs. 35.5%; CC: 36.4% vs. 6.1%) [13]. However, with reference to the HapMap database, our results were concordant with those in a Beijing Chinese Han population (GG: 23.4%, GC: 40.1%, CC: 36.5%). So this discrepancy may be caused by genetic diversity in different ethnic populations. Our results suggest that rs2910164 may not be associated with development of PTC in the Chinese population. In a meta-analysis that summarized the possible association between rs2910164 and cancer risk systematically, no significant associations were found in an overall model of all kinds of cancers [27]. It is possible that rs2910164 may play different roles in cancer susceptibility according to tumor type and ethnicity, and its effect on risk of PTC needs to be studied in other validation populations.

In some retrospective studies, approximately 10% of subjects with nodular goiter in the thyroid glands were found to have incident PTC [28], [29]. But, by now, it is hardly to identify those PTC lesions progressed from BNs, clinically or experimentally. In the current study, 12.7% (96/753) PTC cases had concurrent para-cancer BN. Furthermore, we had found a significant higher frequency of rs2910164 GG homozygote in these patients, compared to 657 PTC without para-cancer BN (28.1% v.s 16.6%). The possible genetic explanation for the association between rs2910164 and simultaneous occurrence of PTC and BN in one thyroid lobe may be that: **1.** PTC and BN are initiated independently but share the same predisposition risk factor (rs2910164) simultaneously, **2.** There is a progression from BN to PTC and rs2910164 is involved in this progression. As summarized in **Tables 2** **,** **4** **and** **5**, our results showed that the rs2910164 GG genotype frequencies in controls, PTC without BN and BN were 18.2%, 16.6% and 16.5%, respectively. These results supported that the rs2910164 G allele does not increase the risk of PTC or BN independently (a similar frequency of GG genotype in each). So, together with the indolent behavior of PTC/BN (lower tumor size, less lymph node metastasis, **Table 4**), we are inclined to the latter hypothesis that some BN are predispose to progress to PTC and remain at an early stage of carcinogenesis and the rs2910164 G allele may play an important role in progression from BN to PTC.

It was useful to find out the PTC cases in patients with thyroid nodules by evaluating gender, age, TSH level, HT status and any other clinical parameters while finding PTC in patients with thyroid nodule is different to identify the cases with BN which may progress to PTC. Results from our study were in agreement with the published results that a high TSH level and Hashimoto thyroiditis were both associated with an increased frequency of PTC in patients with thyroid nodule [21], [22], [23], [24], [25], [26]. However, our results also showed that the rs2910164 genotype and not a high TSH level or Hashimoto thyroiditis contributes to the progression from BN to PTC (**Table 5**). These results were supported by a prospective study which found no increased risk of PTC in Hashimoto’s thyroiditis patients [30].

The potential mechanism of rs2910164 contributing to the progression from BN to PTC is possibly associated with an increased expression of the altered target gene of mature miR-146a caused by the G allele. Jazdzewski et al found that the expression of the mature miR-146a was 2-fold lower in C allele carriers than in G allele carriers in vitro and 3.9-fold lower in CC homozygotes than in GG homozygotes in a series of cell lines [13]. Less than 10% of target genes were shared by the two isoforms produced by the C allele and G allele [15]. All these results present experimental evidence for involvement of the rs2910164 genotype in the carcinogenesis of thyroid cancer.

Some limitations of this study should be acknowledged. Although our study was based on surgical pathology which provided accurate diagnoses of all nodules, we did not include patients who did not have surgery, thus selection bias may exist in patients with BN. Moreover, the mechanism of rs2910164 on thyroid carcinogenesis from BN is based on an association study, and experimental studies to directly confirm the role of rs2910164 are warranted. Although our results should be interpreted with caution, it provided a helpful clue to study the mechanism through what BN progress to a PTC lesion, to elucidate the potential role of rs2910164 in thyroid carcinogenesis and to find out those BNs more predispose to developing a PTC lesion.

### Conclusion

In summary, genotype distributions of rs2910164 were similar between PTC cases, BN patients and healthy controls. Different genotypes were also not associated with focal types or aggressiveness of PTC in the Chinese patients, but GG homozygote carrier had statistically higher portion of PTC/BN. PTC/BN was a special subgroup with relatively indolent behavior and a higher frequency of the GG genotype compared with PTC alone. Multivariate logistic regression analysis showed that the GG homozygote was the only risk factor associated with a possible malignant transformation from BN to PTC, whereas age, gender, serum TSH level and Hashimoto thyroiditis were not associated with this process. As a simple case-control study, our results should be interpreted with caution and more validation populations and experimental evidence are needed in future studies.
